# Mining pathway associations for disease-related pathway activity analysis based on gene expression and methylation data

**DOI:** 10.1186/s13040-017-0127-7

**Published:** 2017-02-01

**Authors:** Hyeonjeong Lee, Miyoung Shin

**Affiliations:** 1 Bio-Intelligence & Data Mining Laboratory, Graduate School of Electronics Engineering, Kyungpook National University, 80, Daehak-ro, Buk-gu, Daegu, 41566 Republic of Korea; 2 School of Electronics Engineering, Kyungpook National University, 80, Daehak-ro, Buk-gu, Daegu, 41566 Republic of Korea

**Keywords:** Biomarker discovery, Gene expression analysis, Pathway association mining, Pathway-set markers, Pathway association network

## Abstract

**Background:**

The problem of discovering genetic markers as disease signatures is of great significance for the successful diagnosis, treatment, and prognosis of complex diseases. Even if many earlier studies worked on identifying disease markers from a variety of biological resources, they mostly focused on the markers of genes or gene-sets (i.e., pathways). However, these markers may not be enough to explain biological interactions between genetic variables that are related to diseases. Thus, in this study, our aim is to investigate distinctive associations among active pathways (i.e., pathway-sets) shown each in case and control samples which can be observed from gene expression and/or methylation data.

**Results:**

The pathway-sets are obtained by identifying a set of associated pathways that are often active together over a significant number of class samples. For this purpose, gene expression or methylation profiles are first analyzed to identify significant (active) pathways via gene-set enrichment analysis. Then, regarding these active pathways, an association rule mining approach is applied to examine interesting pathway-sets in each class of samples (case or control). By doing so, the sets of associated pathways often working together in activity profiles are finally chosen as our distinctive signature of each class. The identified pathway-sets are aggregated into a pathway activity network (PAN), which facilitates the visualization of differential pathway associations between case and control samples. From our experiments with two publicly available datasets, we could find interesting PAN structures as the distinctive signatures of breast cancer and uterine leiomyoma cancer, respectively.

**Conclusions:**

Our pathway-set markers were shown to be superior or very comparable to other genetic markers (such as genes or gene-sets) in disease classification. Furthermore, the PAN structure, which can be constructed from the identified markers of pathway-sets, could provide deeper insights into distinctive associations between pathway activities in case and control samples.

**Electronic supplementary material:**

The online version of this article (doi:10.1186/s13040-017-0127-7) contains supplementary material, which is available to authorized users.

## Background

The problem of finding effective disease markers or signatures is very important for the successful diagnosis, treatment, and prognosis of complex diseases such as cancers. So far, many studies have worked on identifying disease-related markers with a variety of biological resources, such as gene expression profiles [1–8], protein-protein interactions [9–11], pathway databases [12, 13], and so on. However, most of them focused on examining the markers of individual genes or gene-sets (i.e., pathways) as disease signatures. For example, such conventional methods like t-tests, fold change, and signal-to-noise ratio were used to find differentially expressed genes [14–16] without regard to gene associations or other biological information. On the other hand, some studies explored gene associations for disease marker findings. For example, Maulik et al. [17] proposed a rule mining method that employs biclustering analysis to discover interesting relationships among genes under certain conditions. Giugno et al. [18] generated association rules between gene expression intervals for classification. Other researchers performed pathway-based analyses to consider the predefined gene-sets (i.e., pathways) as disease markers [19–25]. Kim et al. [22] introduced pathway-based markers by combining gene-set enrichment analysis with support vector machine. Lee et al. [23] identified core genes in pathways for disease classification in pathway level.

Although such methods were shown to be very useful in many applications, they may not be enough to fully understand disease mechanisms. Moreover, multiple candidate pathways often share some genes, so it leads to forming complex interconnections among pathways while making analysis and interpretation complicated. For example, when there are common genes in multiple candidate pathways, they might be considered to be associated with each other. However, even when multiple candidate pathways do not share genes, they may be mutually associated or active together. Thus, it seems worthwhile to investigate the associations among the activities of significant pathways differentially shown in case and control samples, as potential disease signatures.

**Fig. 1:**
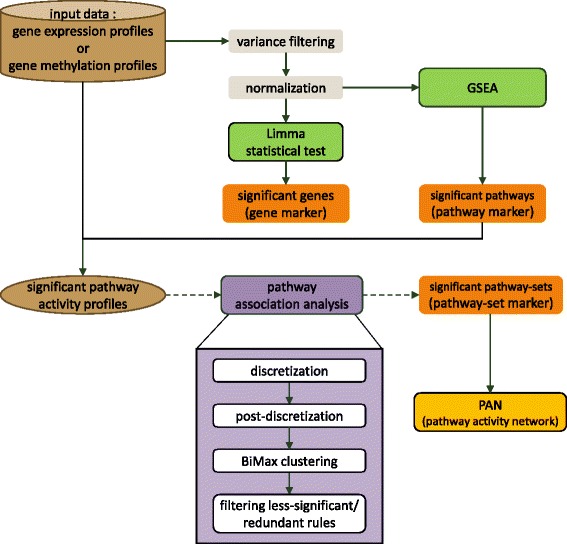
The overall workflow for pathway association mining approach in our study

In this study, our aim is to identify the sets of pathways (i.e., pathway-sets) having associations in the activities observed from gene expression profiles (or DNA methylation data), especially shown differentially in case and control samples. For this purpose, we utilized an association rule mining approach to find active pathway-sets that are shown distinctively and frequently in each class of samples (case or control). This was done based on the assumption that the set of pathways often being active together in gene expression (or methylation) profiles could have some interesting biological relationships among them. For the discovery of interesting pathway-sets, we conducted gene-set enrichment analysis (GSEA) [26] to identify significant (active) pathways and derived pathway activity profiles from gene expression (or methylation) data. Then, we applied the BiMax clustering on the pathway activity profiles to detect clusters of co-activated pathways frequently shown in class samples. In the end, interesting pathway-sets were found by taking the significant sets of co-activated pathways that satisfy a certain threshold of significance measures. For the evaluation of the identified pathway-sets, we used them as disease markers for classification and analyzed the performance. Besides, we aggregated the inferred pathway-sets in each class into a pathway activity network (PAN) in which nodes represent significant active pathways and edges represent interesting associations between the pathways. This PAN facilitates the visualization of differential pathway associations shown in case and control samples. Figure 1 summarizes the overall workflow of our pathway association mining approach used in this study.

## Results and discussion

For experiments, we utilized two publicly available cancer datasets in this study. GSE15852 [27] (Dataset 1) is the gene expression data about breast cancer, which includes 86 samples (43 controls, 43 cases) of 12,500 probes. GSE31699 [28] (Dataset 2) is the dataset for uterine leiomyoma cancer that contains mRNA expression profiles of 48,803 probes in 16 uterine leiomyoma tumor (LM) samples and 16 normal myometrial (MM) samples and DNA methylation profiles of 27,578 probes in18 uterine LM samples and 18 normal MM samples.

### Identification of pathway-set markers in breast tumors and paired normal tissues

**Fig. 2:**
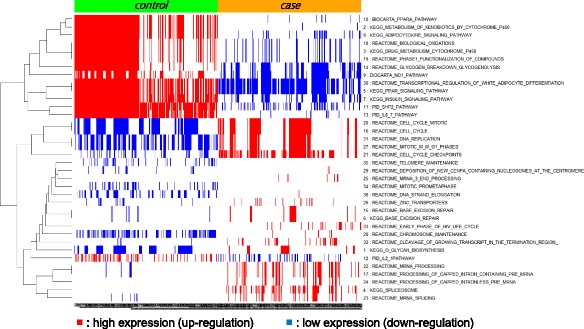
The heatmap of 500 pathway-set markers identified by our proposed approach from Dataset 1

In breast cancer dataset (Dataset 1), we first identified 36 significant pathways by GSEA with FDR q-value < 0.05. Then, we produced the activity profiles of these pathways and utilized them to produce pathway-sets satisfying minimum support of 25%. (See the Methods section for details) Out of these pathway-sets, we have chosen top 250 significant pathway-sets in each class as disease markers for further analyses. Figure 2 shows the heatmap of our 500 pathway-set markers in which the rows represent 36 significant pathways and the columns represent the activities of 500 pathway-sets. Also up-regulated pathways are shown in red and down-regulated pathways are shown in blue. From this figure, we can clearly find the distinctive feature of activities in the chosen pathway-sets between case and control classes. The details of 36 selected pathways and top 10 interesting pathway-sets that we identified are given in Additional file 1.

### Identification of pathway-set markers in uterine leiomyoma and matched myometrial tissues

**Fig. 3:**
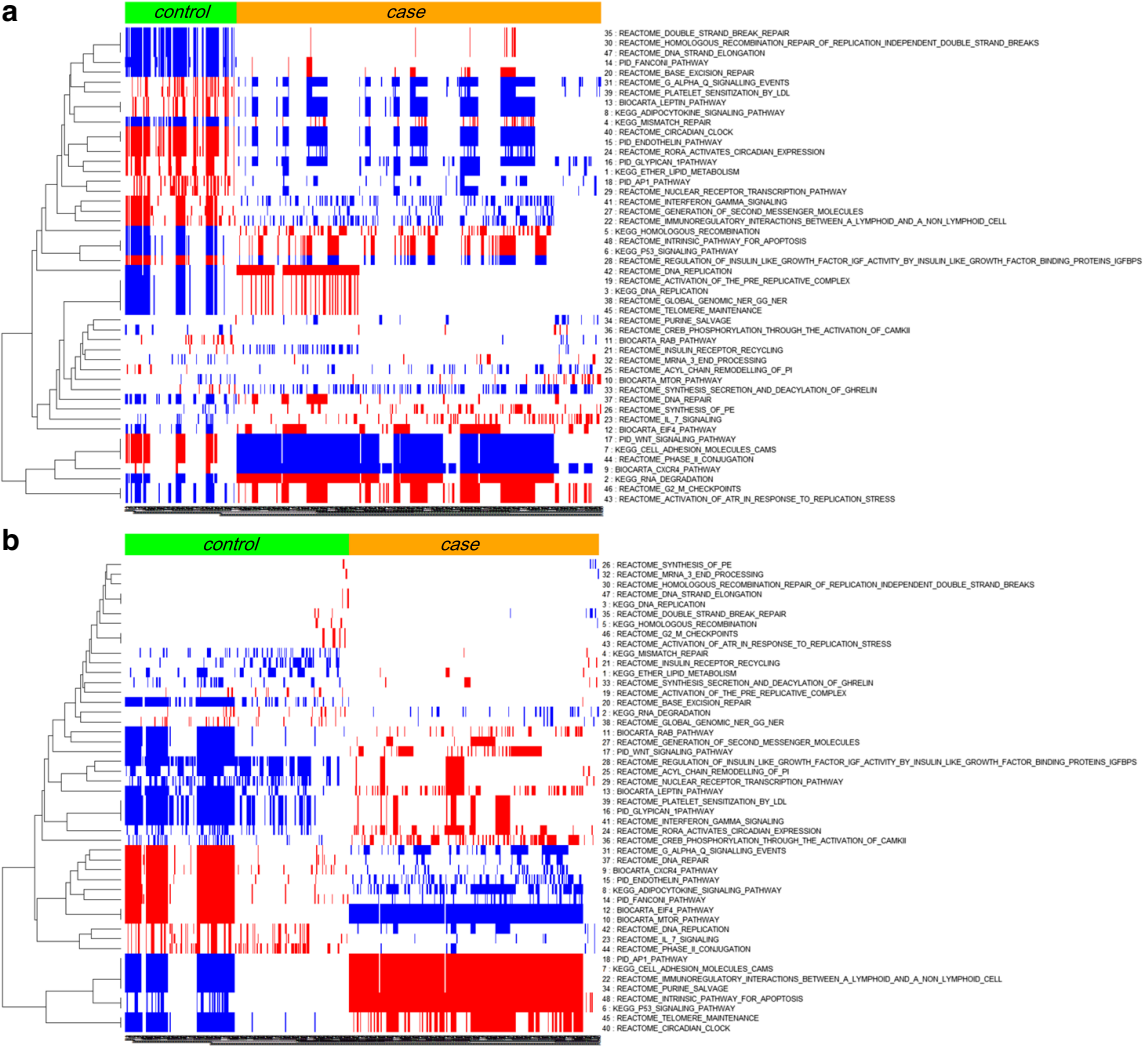
The heatmap of all the interesting pathway-sets obtained from (**a**) gene expression data and (**b**) methylation data. **a** includes 116 pathway-sets in control and 380 in case while (**b**) includes 260 pathway-sets in control and 290 in case, respectively

**Fig. 4:**
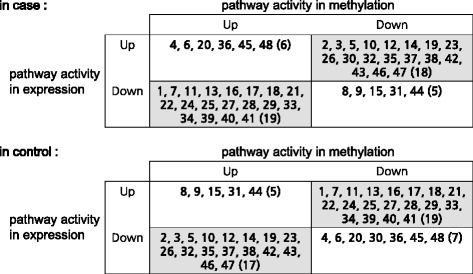
Relationship between pathway activities shown in gene expression and methylation data

In uterine leiomyoma cancer dataset (Dataset 2), we first identified 24 pathways as differentially expressed (DE) pathways from gene expression profiles and 25 pathways as differentially methylated (DM) pathways from methylation data, by using GSEA. Then, we chose the union of all these pathways (48 pathways) as significant pathways and used them to detect interesting pathway-sets from gene expression data and methylation data, respectively. The details of 48 pathways are given in Additional file 2. All the interesting pathway-sets obtained from gene expression data and methylation data are shown in Fig. 3(a) and (b), respectively. From this figure, it is seen that the activities of our chosen pathway-sets are clearly distinguishable between case and control classes. Also, from these pathway-sets, the relationships between pathway activities shown in gene expression and methylation data were analyzed. Interestingly, out of 48 pathways, we could find an inverse relationship between pathway activities shown in gene expression and methylation data from 37 pathways in case and 36 pathways in control, which are given in Fig. 4.

### Evaluation of pathway-set markers with disease classification

For disease classification, three different types of genetic markers (including the markers of genes/pathways/pathway-sets) were used and compared in terms of the sensitivity, specificity, and accuracy. In the breast cancer dataset (Dataset 1), 67 differentially expressed genes were identified as gene markers using Limma with *p*-value < 0.05, whereas 36 pathway markers were found by GSEA. As pathway-set markers, we used 500 pathway-sets chosen in earlier stage. For classification with pathway-set markers, we derived the activities of pathway-sets from pathway activity profiles in the similar way to define pathway activities from gene expression profiles. Table 1 summarizes our classification results averaged over 8 repetitions with three types of markers (genes, pathways, pathway-sets) in Dataset 1. According to this table, overall, the pathway-set markers were superior or very comparable to other markers (genes, pathways) regardless of classification method.

**Table 1:** Comparison of classification performance with three types of markers in the breast cancer dataset

Classifiers	Markers	Sensitivity	Specificity	Accuracy
kNN	genes	93.6 (2.06)	82.56 (3.29)	88.08 (1.94)
	pathways	92.44 (1.08)	89.53 (1.76)	90.99 (1.2)
	pathway-sets	97.09 (2.06)	90.7 (1.24)	93.9 (1.03)
RF	genes	87.21 (1.76)	87.79 (1.08)	87.5 (0.82)
	pathways	93.31 (2.62)	92.73 (1.49)	93.02 (1.08)
	pathway-sets	93.31 (2.62)	92.73 (1.49)	93.31 (1.03)
SVM	genes	86.63 (1.08)	86.63 (2.41)	86.63 (1.52)
	pathways	93.9 (1.73)	91.28 (1.08)	92.59 (1.23)
	pathway-sets	91.57 (3.27)	92.44 (1.08)	92.01 (1.91)
Naïve Bayes	genes	86.63 (1.08)	86.63 (2.41)	86.63 (1.52)
	pathways	93.9 (1.73)	91.28 (1.08)	92.59 (1.23)
	pathway-sets	91.57 (3.27)	92.44 (1.08)	92.01 (1.91)

For uterine leiomyoma cancer dataset (Dataset 2), we identified 276 differentially expressed genes (DEGs) from gene expression and 1370 differentially methylated genes (DMGs) from methylation data using Limma with *p*-value <0.001, respectively. Among them, we selected 28 common genes between 276 DEGs and 1370 DMGs as gene markers. For pathway and pathway-set markers, we used the 48 pathways and 496 pathway-sets identified earlier, respectively. Table 2 shows our classification results in Dataset 2, which reveals that all the markers are comparable in classification performance.

**Table 2:** Comparison of classification performance with three types of markers in the uterine leiomyoma cancer dataset

Classifiers	Markers	Sensitivity	Specificity	Accuracy
kNN	genes	93.75	92.97 (2.21)	93.36 (1.1)
	pathways	92.97 (2.21)	93.75	93.36 (1.1)
	pathway-sets	93.75	93.75	93.75
RF	genes	90.62 (3.34)	93.75	93.36 (1.1)
	pathways	92.97 (2.21)	92.97 (2.21)	92.19 (1.45)
	pathway-sets	93.75	92.97 (2.21)	93.36 (1.1)
SVM	genes	93.75	92.19 (2.89)	92.97 (1.45)
	pathways	92.97 (2.21)	93.75	93.75
	pathway-sets	93.75	93.75	93.75
Naïve Bayes	genes	93.75	92.19 (2.89)	92.97 (1.45)
	pathways	93.75	93.75	93.75
	pathway-sets	93.75	93.75	93.75

In addition, we performed paired *t*-test to support the claim that the difference in classification accuracies between biomarkers is significant, as in Table 3. From this table, it is found that pathway-set markers are significantly superior to gene markers in Dataset 1 for all 4 classification methods, while showing no significant difference from gene markers in Dataset 2 except kNN. Also, there was no significant difference between pathway-set and pathway markers except in kNN. That is, pathway-set markers are superior or comparable to other biomarkers (genes, pathways) in classification performance.

**Table 3:** Statistical significance (*p*-values) of the difference in classification accuracies between biomarkers in Dataset 1 and Dataset 2

	Dataset 1		Dataset 2	
	pathway-set vs gene	pathway-set vs pathway	pathway-set vs gene	pathway-set vs pathway
kNN	3.316504e-05	0.0005898392	0.01994213	NaN
RF	1.241385e-06	0.3506167	0.1035517	NaN
SVM	5.368579e-05	0.4699636	NaN	NaN
Naïve Bayes	5.368579e-05	0.4699636	NaN	NaN

### Distinctive features of pathway activity networks in case and control samples

To have better understanding of the identified pathway-set markers, we constructed the pathway activity network (PAN) (See the Method section for details). Based on our pathway-sets, two different PANs were generated that demonstrate differential pathway associations appeared in case and control samples, respectively. By doing so, we could easily capture the distinctive features of pathway activities and their associations shown in class samples. In the PAN, the thickness of an edge is proportional to the relative frequency of pathway associations shown in pathway-set markers of class samples. The size of a node is proportional to the relative significance of corresponding pathway in the sense of how much distinguishable its activity level is between case and control samples. Thus, as pathway is more significant, its node size would be larger. Also, the color of a node indicates the activity type of its corresponding pathway in terms of expression changes, i.e., the node is displayed in red when its corresponding pathway is up-regulated under the class condition (case/control) or in blue when it is down-regulated.

**Fig. 5:**
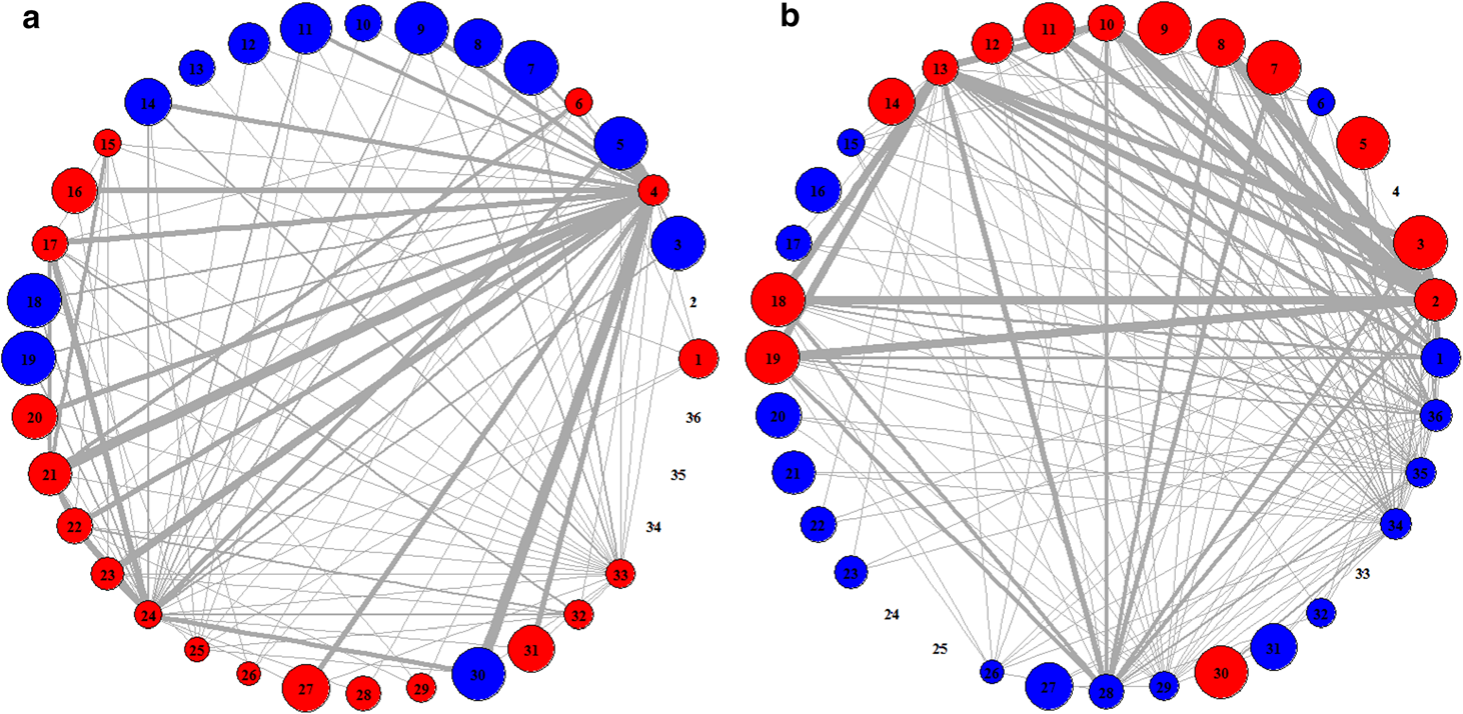
Differential patterns of PANs in case (*left*) and control (*right*) classes from Dataset 1

For Dataset 1, we obtained the two PANs shown in case and control samples, as in Fig. 5. They clearly revealed distinctive features of pathway activities each in case and control classes. In particular, Fig. 5(a) and (b) show the distinguishable signature of pathway associations existing only in case PAN and only in control PAN, respectively. For example, in Fig. 5(a), it is seen that pathway 4 (spliceosome) has significant associations with many other pathways (including pathways 14, 16, 17, 20, 21, 22, 23, 27, 30, and etc.), indicating that these associations are observed only in case samples while not in control samples as in Fig. 5(b). In particular, it is interesting that even if pathway 4 (spliceosome) itself is relatively not that significant in pathway-level analysis, our pathway-set analysis could newly discover the significance of pathway 4 in the associations with many other pathways. Thus, such distinctive features of pathway associations shown in case samples may be possibly good disease signature itself or vital clues to the disease signature. To verify the biological significance of pathway associations shown over the PAN, we searched for literatures and found some interesting evidences that pathway 4 (spliceosome) is related to the cause of breast cancer [29]. Moreover, pathway 30 (transcriptional regulation of white adipocyte differentiation) that is significantly associated with pathway 4 over the PAN is known to have relevance to breast cancer [30, 31].

**Fig. 6:**
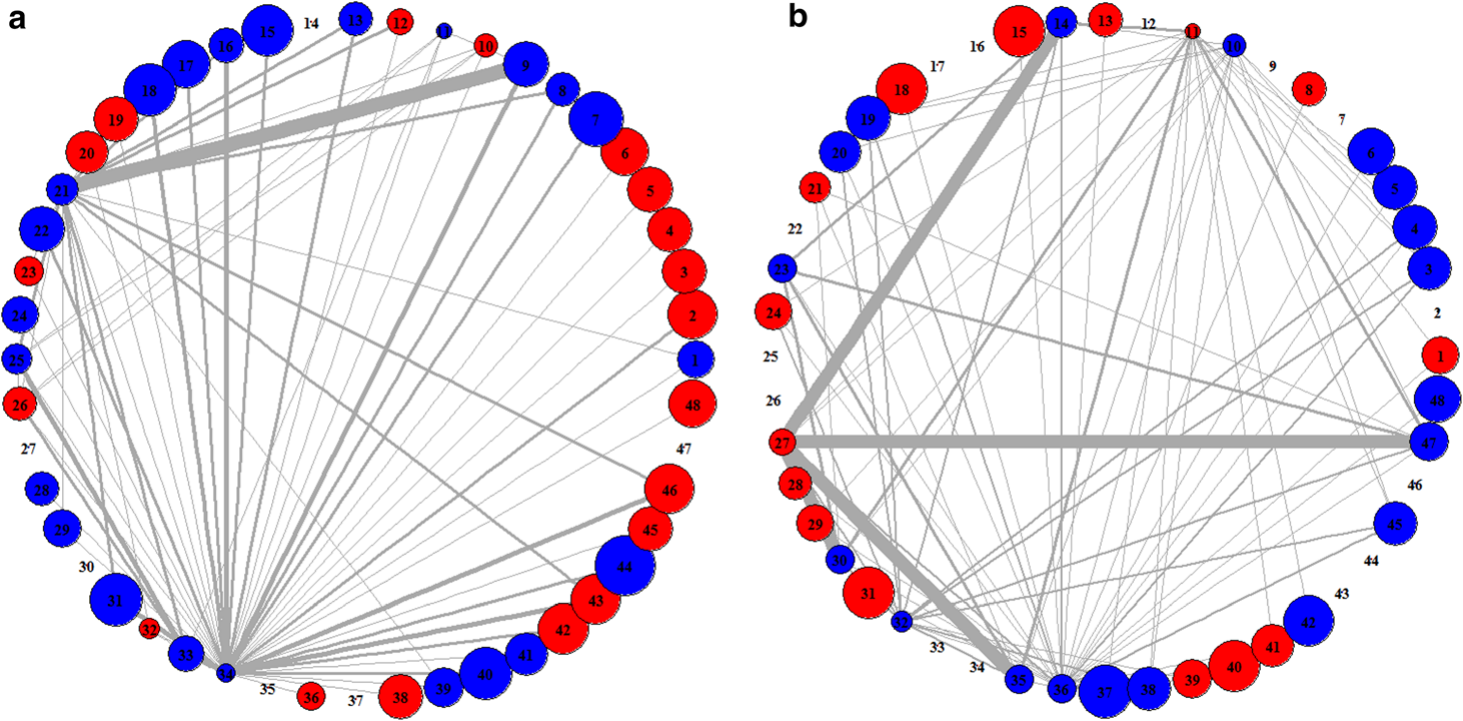
PANs differentially shown in case (*left*) and control (*right*) class obtained from mRNA expression in Dataset 2

**Fig. 7:**
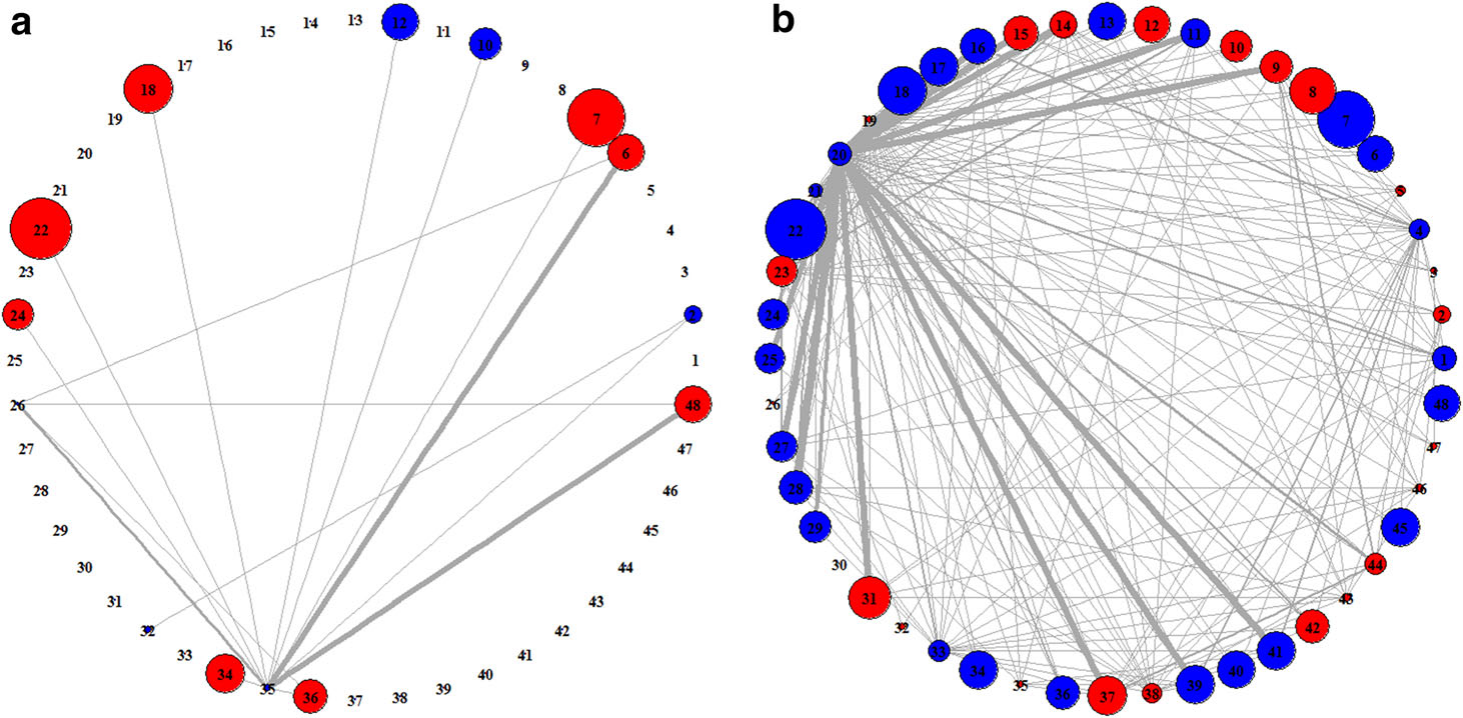
PANs differentially shown in case (*left*) and control (*right*) classes obtained from DNA methylation in Dataset 2

For Dataset 2, we obtained the PANs shown in case and control samples of gene expression data, which are given in Fig. 6. Also, the PANs shown in case and control samples of methylation data are given in Fig. 7. In Fig. 6, we can see that pathway 9 (CXCR4 pathway) is significantly associated with pathway 21 (insulin receptor recycling) only in case samples. Whereas, in Fig. 7, it is observed that pathway 35 (double strand break repair) has some interesting associations with pathway 6 (P53 signaling pathway) and pathway 48 (intrinsic pathway for apoptosis) only in case samples.

## Conclusions

In this study, we have focused on identifying interesting pathway-sets that indicate the distinctive features of the activities of significant pathways and their associations shown in case and control samples. These pathway-sets were found by considering sets of significant pathways frequently activated together over class samples. For this purpose, we utilized an association rule mining approach that employs the BiMax biclustering method to detect pathway associations with discretized pathway activity profiles. Even if the BiMax method is relatively faster and more time-efficient than traditional approaches like Apriori algorithm that often create too many rules and is time-consuming, it still has the practical problem such that the number of clusters tends to increase drastically as the number of input features (i.e., the number of significant pathways) increases, leading to the radical increase of corresponding pathway-sets. To handle this situation, we prioritized the identified pathway-sets using rule interestingness measures and selected top-*n* most significant pathway-sets. The chosen pathway-sets performed superior or very comparable to other biomarkers in disease classification. Moreover, by constructing pathway activity network from the identified pathway-sets, we could gain deeper insights into pathway associations differentiating between case and control samples. However, since our experiments were conducted with only one disease and one kind of control types, the results we obtained in this study may have some limitation. To strengthen the prominence of our approach, it will be necessary to perform additional experiments with wide selection of data, including multiple cancer and tissue types. Although our work may not cover all aspects of biomarker identification issues, the methodology used in this study could be still applicable to discover interesting biomarkers for other diseases, as well as to underpin the biological mechanisms of target diseases. As future works, we plan to perform pathway association studies with multiple cancer types. We also plan to work on identifying disease-specific markers using normal samples of multiple tissue types in near future.

## Methods

### Data preparation

In the gene expression (or methylation) data, when there are multiple probes corresponding to gene, we used their averaged expression values as an expression value of the gene. Also, such genes that the variances of their expression values are lower than a certain threshold were excluded from the data, and zero-mean normalization was applied to adjust different scales of genes into a common scale. We used MSigDB C2 curated data to define candidate pathways and found significant (active) pathways among the candidates by performing GSEA with gene expression or methylation data, in which two different methods (i.e., SNR and *t*-test) were used to find differentially expressed genes. From two GSEA results obtained by SNR and *t*-test, respectively, common pathways were considered as significant pathways.

In case of the uterine leiomyoma cancer datasets (Dataset 2), since it contains gene expression profiles and DNA methylation data, significant pathways were finally chosen by taking the union of significant pathways found from gene expression data and from DNA methylation data, respectively.

### Finding pathway-set markers by pathway association analysis

**Fig. 8:**
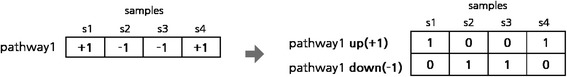
An example of 2*n*-bit binarized pathway activity vector produced from *n*-bit discretized pathway activity vector

For pathway association analysis, we first obtained pathway activity profiles from gene expression (or methylation) data by adapting the method used in [23] and discretized them by assigning either −1 (lowly expressed, down-regulated) or +1 (highly expressed, up-regulated) to each value of pathway activity. Then, these discretized pathway activity data of +1’s and -1’s were converted into the binarized data of 0’s and 1’s for biclustering analysis. Specifically, for each *n*-dimensional pathway activity vector of +1’s and/or -1’s, we produced a 2*n*-dimensional binary vector of 1’s and 0’s by assigning first *n* bits to the binarization of up-regulated data (or hyper-methylation data) and other *n* bits to the binarization of down-regulated data (or hypo-methylation data), as in Fig. 8. Next, with the binarized pathway activity data, we performed the BiMax biclustering analysis to generate such clusters that each cluster should contain at least 2 pathways and as many samples as satisfying a minimum support threshold. Here the minimum support specifies the minimum size of samples included in each cluster. For example, if minimum support is 25%, it means that each cluster should have at least 25% of class samples or more. As a minimum support threshold increases, the number of generated clusters would decrease while they would have higher support.

**Fig. 9:**
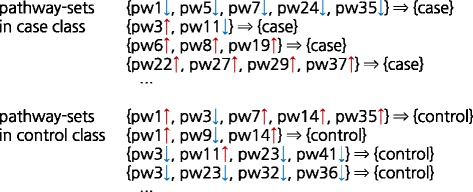
Example of interesting pathway-sets in the rule form for each class, in which an *upward pointing arrow* (or *downward pointing arrow*) indicates up-regulation (or down-regulation) of each pathway

Once all clusters were found for case and control samples, respectively, we examined each of them to generate association rules. Prior to rule generation, we calculated the confidence and the lift of each cluster for evaluation, and eventually produced the association rules only for the clusters that satisfy the thresholds of confidence ≥0.8 and lift >1. If a certain cluster were found in the both classes, two association rules are considered; one for the case and the other for the control. Out of these two rules, the less-significant rules are eliminated. Then, from all the produced association rules, we extracted interesting pathway-sets. Figure 9 illustrates an example of interesting pathway-sets in the rule form of {pathway-set} ⇒ {class}, where the constituent pathways in a pathway-set are expressed as being up-regulated (↑) or down-regulated (↓).

### Disease classification with pathway-set markers

From the pathway-set markers, we obtained pathway-set activity profiles by applying the method adapted from Lee et al. [23] to pathway activity profiles, and used them for classification. That is, the activity of pathway-set was defined by combining the activities of pathways in such a way to have the most discriminative power in the pathway-set. To calculate discriminant power, we used a statistical test Limma that performs well regardless of sample size and data distribution.

For classification with pathway-set markers, we employed four classification methods, including k-nearest neighbor (KNN), support vector machine (SVM), random forest (RF), and naïve Bayesian classifiers. This task is to predict a target class (control or case) of a given sample from the distinctive signature shown in identified pathway-set markers. For performance evaluation, we used 4-fold cross-validation with 8 repetitions in which each repetition is for random selection in data partitioning, and compared the averaged classification results over 8 repetitions in terms of sensitivity, specificity, and accuracy. We also conducted paired *t*-test between the accuracies of biomarkers (genes, gene-sets, and pathway-sets) and examined if the difference between classification accuracies is statistically significant.

### Construction of pathway activity network from pathway-set markers

For better understanding of the identified pathway-set markers, we constructed pathway activity network (PAN) in which the nodes represent significant (active) pathways, the edges represent the associations between active pathways. The thickness of an edge represents the relative frequency of pathway associations shown in the identified pathway-set markers. Also, the size of a node represents the relative significance of pathway that is defined as –log2 (*p*-value) when *p*-value is a statistical significance of the difference in its activity level between case and control classes. The size of a node represents the relative significance of corresponding pathway in the sense of how much distinguishable its activity level is between case and control samples. The PAN can be drawn separately for each class (case or control), which shows distinctive features of pathway activities and their associations in case or control samples.

## Additional files

Additional file 1: (a). Significant pathways in Dataset 1. (b). Top 10 interesting pathway-sets obtained from Dataset 1. (PDF 199 kb)
Additional file 2: Significant pathways in Dataset 2 and 3. (PDF 103 kb)

## Abbreviations

BiMax: Binary inclusion-maximal biclustering algorithm
GSEA: Gene set enrichment analysis
MSigDB: The molecular signatures database
