# Supplementary material for: Mining pathway associations for disease-related pathway activity analysis based on gene expression and methylation data
Source: BioData Min. 2017 Feb 1;10:3. doi: 10.1186/s13040-017-0127-7 (PMC5286825; doi:10.1186/s13040-017-0127-7)
Supplement: Additional file 1: — (a). Significant pathways in Dataset 1. (b). Top 10 interesting pathway-sets obtained from Dataset 1. (PDF 199 kb) [file 13040_2017_127_MOESM1_ESM.pdf]

Additional file 1.

1-(a). Significant pathways in Dataset 1

[1] KEGG O-Glycan biosynthesis

[2] KEGG metabolism of xenobiotics by cytochrome P450

[3] KEGG drug metabolism - cytochrome P450

[4] KEGG spliceosome

[5] KEGG PPAR signaling pathway

[6] KEGG base excision repair

[7] KEGG insulin signaling pathway

[8] KEGG adipocytokin signaling pathway

[9] BIOCARTA no.1 pathway

[10] BIOCARTA PPARa pathway

[11] PID SHP-2 pathway

[12] PID IL-21 pathway

[13] PID IL-67 pathway

[14] REACTOME glycogen breakdown (glycogenolysis)

[15] REACTOME base excision repair

[16] REACTOME cell cycle

[17] REACTOME processing of capped intron-containing pre-mRNA

[18] REACTOME biological oxidations

[19] REACTOME phase1 functionalization of compounds

[20] REACTOME cell cycle mitotic

[21] REACTOME cell cycle checkpoints

[22] REACTOME mRNA processing

[23] REACTOME mRNA splicing

[24] REACTOME processing of capped intronless pre-mRNA

[25] REACTOME mRNA 3’-end processing

[26] REACTOME Zinc transporters

[27] REACTOME mitotic M-M/G1 phases

[28] REACTOME chromosome maintenance

[29] REACTOME deposition of new CENPA-containing nucleosomes at the centromere

[30] REACTOME transcriptional regulation of white adipocyte differentiation

[31] REACTOME DNA replication

[32] REACTOME cleavage of growing transcript in the termination region

[33] REACTOME early phase of HIV life cycle

[34] REACTOME mitotic prometaphase

[35] REACTOME telomere maintenance

[36] REACTOME DNA strand elongation

1-(b). Top 10 interesting pathway-sets obtained from Dataset 1

{pw1↓, pw2↑, pw3↑, pw5↑, pw7↑, pw8↑, pw9↑, pw10↑, pw11↑, pw13↑, pw14↑, pw16↓, pw18↑, pw19↑, pw20↓, pw30↑} ⇒ {control}

{pw2↑, pw3↑, pw5↑, pw7↑, pw8↑, pw10↑, pw11↑, pw12↑, pw13↑, pw14↑, pw16↓, pw18↑, pw19↑, pw20↓, pw30↑} ⇒ {control}

{pw2↑, pw3↑, pw5↑, pw7↑, pw8↑, pw9↑, pw10↑, pw11↑, pw13↑, pw14↑, pw18↑, pw19↑, pw27↓, pw30↑, pw31↓} ⇒ {control}

{pw2↑, pw3↑, pw5↑, pw7↑, pw8↑, pw9↑, pw10↑, pw11↑, pw12↑, pw13↑, pw14↑, pw18↑, pw19↑, pw30↑} ⇒ {control}

{pw2↑, pw3↑, pw5↑, pw7↑, pw8↑, pw10↑, pw11↑, pw13↑, pw14↑, pw18↑, pw19↑, pw28↓, pw30↑} ⇒ {control}

{pw5↓, pw16↑, pw20↑, pw30↓} ⇒ {case}

{pw12↓, pw16↑, pw20↑} ⇒ {case}

{pw5↓, pw21↑, pw30↓} ⇒ {case}

{pw5↓, pw16↑, pw20↑, pw21↑, pw27↑, pw30↓, pw31↑} ⇒ {case}

{pw5↓, pw9↓, pw16↑, pw20↑, pw30↓} ⇒ {case}
