# Supplementary material for: Mining pathway associations for disease-related pathway activity analysis based on gene expression and methylation data
Source: BioData Min. 2017 Feb 1;10:3. doi: 10.1186/s13040-017-0127-7 (PMC5286825; doi:10.1186/s13040-017-0127-7)
Supplement: Additional file 2: — Significant pathways in Dataset 2 and 3. (PDF 103 kb) [file 13040_2017_127_MOESM2_ESM.pdf]

Additional file 2.

Significant pathways in dataset 2 and 3

[1] KEGG ether lipid metabolism

[2] KEGG RNA degradation

[3] KEGG DNA replication

[4] KEGG mismatch repair

[5] KEGG homologous recombination

[6] KEGG P53 signaling pathway

[7] KEGG cell adhesion molecules (CAMs)

[8] KEGG adipocytokine signaling pathway

[9] BIOCARTA CXCR4 pathway

[10] BIOCARTA mTOR pathway

[11] BIOCARTA Rab pathway

[12] BIOCARTA eIF4 pathway

[13] BIOCARTA leptin pathway

[14] PID fanconi pathway

[15] PID endothelin pathway

[16] PID glypican 1 pathway

[17] PID WNT signaling pathway

[18] PID AP1 pathway

[19] REACTOME activation of the pre-replicative complex

[20] REACTOME base excision repair

[21] REACTOME insulin receptor recycling

[22] REACTOME immunoregulatory interactions between a lymphoid and a non-lymphoid cell

[23] REACTOME IL-7 signaling

[24] REACTOME RORA activates circadian expression

[25] REACTOME Acyl chain remodeling of PI

[26] REACTOME synthesis of PE

[27] REACTOME generation of second messenger molecules

[28] REACTOME regulation of insulin like growth factor (IGF) activity by insulin like growth factor binding proteins (IGSBPs)

[29] REACTOME nuclear receptor transcription pathway

[30] REACTOME homologous recombination repair of replication independent double strand breaks

[31] REACTOME G alpha (q) signalling events

[32] REACTOME mRNA 3’-END processing

[33] REACTOME synthesis secretion and deacylation of ghrelin

[34] REACTOME purine salvage

[35] REACTOME double strand break repair

[36] REACTOME CREB phosphorylation through the activation of CAMKII

[37] REACTOME DNA repair

[38] REACTOME global genomic NER (GG-NER)

[39] REACTOME platelet sensitization by LDL

[40] REACTOME circadian clock

[41] REACTOME interferon gamma signaling

[42] REACTOME DNA replication

[43] REACTOME activation of ATR in response to replication stress

[44] REACTOME phase II conjugation

[45] REACTOME telomere maintenance

[46] REACTOME G2/M checkpoints

[47] REACTOME DNA strand elongation

[48] REACTOME intrinsic pathway for apoptosis
